# Predictive factors of renal function after robot-assisted partial nephrectomy in clinical T1b tumors

**DOI:** 10.1007/s11701-024-01848-3

**Published:** 2024-04-02

**Authors:** Ryohei Yamamoto, Kazuyuki Numakura, Mizuki Kobayashi, Taketoshi Nara, Mitsuru Saito, Shintaro Narita, Tomonori Habuchi

**Affiliations:** Department of Urology, Akita University Graduate School of Medicine, 1-1-1 Hondo, Akita, 010-8543 Japan

**Keywords:** Robot-assisted partial nephrectomy, Renal function, Clinical T1b renal cell carcinoma, RENAL nephrometry score

## Abstract

Robot-assisted partial nephrectomy (RAPN) has been shown to be a safe and effective method for treatment of small renal tumors, including clinical T1b renal cell carcinoma (RCC); however, the impact of RAPN for cT1b renal tumors on renal function is not well understood. In this retrospective study, 50 patients who underwent RAPN for cT1b renal tumors were evaluated for pre- and post-operative renal function and perioperative clinical factors. Renal function was assessed using the estimated glomerular filtration rate (eGFR) at baseline and on postoperative days (POD) 1, 7, 30, and 180.A significant renal functional decline was defined as ≥ 15% reduction in eGFR at POD180 compared with eGFR at baseline. Logistic regression analyses were used to identify risk factors for renal function decline, including age, sex, RENAL nephrometry score, operative time, and estimated blood loss. The median patient age was 62 years, and the median tumor diameter and RENAL nephrometry score were 44 mm (IQR 43–50) and 8 (IQR 7–9), respectively. Of these patients, 16 (36%) showed a significant renal functional decline at POD 180. In the multivariate analysis, the L component of the RENAL nephrometry score and an estimated blood loss of 200 mL or more were identified as significant risk factors for renal functional decline. These findings suggest that the preoperatively definable L component of the RENAL nephrometry score and intraoperative blood loss, which may be modifiable factors, play significant roles in post-RAPN renal function decline.

**Supplementary Information:**

The online version contains supplementary material available at 10.1007/s11701-024-01848-3.

## Introduction

RCC is a common malignancy that accounts for approximately 2–3% of all adult cancers [1]. Radical nephrectomy (RN) has traditionally been the standard treatment of RCC. However, this procedure can significantly decrease renal function and increase the risk of chronic kidney disease (CKD) [2]. Partial nephrectomy (PN) has emerged as a viable alternative to RN because it can preserve kidney function while effectively treating clinically localized RCC for cancer control [3]. Moreover, with the increasing adoption of robot-assisted surgical systems, robot-assisted partial nephrectomy (RAPN) has been now recognized as standard treatment strategy for small renal tumors because it has been shown to be safe and oncologically efficient, while being associated with efficient preservation of renal function [4–6]. Previous studies have indicated no significant difference in cancer-specific survival between PN and RN for cT1a and cT1b RCC [5, 7]. Consequently, its applicability was expanded to include cT1b or larger RCCs. However, little is known about renal function after RAPN for cT1b RCC despite it being chosen as the treatment of choice for kidney preservation.

This study aimed to evaluate the effect of clinical factors on renal function after RAPN. Our findings may help clinicians better assess the risks and benefits of RAPN and share decision-making regarding treatment options for patients with cT1b RCC.

## Materials and methods

Fifty patients who underwent RAPN for cT1b renal tumor of the 262 patients who underwent RAPN for renal tumors between November 2017 and September 2022 at Akita University Hospital were included in this retrospective study. Data were collected from the medical records. Relevant information, such as demographic data, clinical parameters, and treatment details was extracted for each participant. The tumor size was measured, and the RENAL nephrometry score was assessed according to a preoperative dynamic CT scan with early arterial and late excretory phase images. Renal function was assessed using the estimated glomerular filtration rate (eGFR) based on the estimation equation for Japanese patients with CKD [8] and measured at baseline and postoperative day (POD) 1, 7, 30, and 180.

A significant renal functional decline was defined as a 15% or more reduction in eGFR at POD180 compared with the eGFR at baseline [9]. Postoperative complications were evaluated using the Clavien–Dindo classification version 4.0. A single surgeon (T.H.) performed all procedures using the Da Vinci Surgical System Si or Xi. Based on the tumor size and location, we determined whether to use a retroperitoneal or transperitoneal surgical approach in each case. We maintained a pneumoperitoneum pressure of 10 mm Hg and increased it to 15 mm Hg during tumor resection. The resected line of the tumor was determined using ultrasonography and marked with a monopolar diathermic knife before vessel clamping. In cases involving tumors close to the renal hilum, both the renal artery and vein were clamped using a Reliance Bulldog clamp (ACP, JAPAN), whereas in other cases, only the renal artery was clamped before resection. Renal tumors were divided with a 5 mm parenchymal margin. Both inner- and outer-layer renorrhaphies were performed using 2–0 STRATAFIXTM Spiral PDSTM Plus (Ethicon, USA). After finishing the inner suture, the clamp was released (early unclamping technique [10, 11]), and the outer renorrhaphy was sutured using the sliding hemo-lock technique Surgical specimens were removed using an ENDOPOUCHTM RETRIEVERTM Specimen bag (AutoSuture, UK) via a camera port. A trifecta was defined as warm ischemia time (WIT) ≤ 25 min, negative surgical margins, and absence of Clavien–Dindo grade 3 or more complications.

All statistical analyses were performed using SPSS ver. 28. Continuous variables are reported as median and interquartile range (IQR).

The significance level was set at p < 0.05 all analyses. To compare variables between groups, Mann–Whitney U tests were performed for continuous variables. Univariate and multivariate analyses for the presence of a significant decline in renal function (> 15%) were performed using the multiple logistic regression analysis and odds ratios (ORs) were estimated with 95% confidence intervals (CIs).

## Results

Of the 50 patients enrolled in the study, 72% were male, and the median age was 62 years (IQR: 55–70). The median body mass index was 23.7 kg/m^2^ (IQR: 21.4–26.0). The median tumor diameter and RENAL nephrectomy score were 44 mm (IQR: 43–50) and 8 (IQR: 7–9), respectively. The median eGFR at baseline was 70 mL/min/1.73 m^2^ (IQR: 60–78) (Table 1). The common comorbidities were hypertension (48%), diabetes mellitus (18%), and hyperlipidemia (36%).

**Table 1:** Demographics of patients

	n = 50
Age, years (median [IQR])	62 (55−70)
Sex, n (%)	
Male	36 (72)
Female	14 (28)
BMI, kg/m^2^ (median [IQR])	23.7 (21.4−26.0)
Diabetes mellitus, n (%)	9 (18)
Hypertension, n (%)	24 (48)
Hyperlipidemia, n (%)	18 (36)
Laterality, n (%)	
Right	23 (46)
Left	27 (54)
Tumor diameter, mm (median [IQR])	44 (43−50)
RENAL nephrometry score (median [IQR])	8 (7−9)
R component (median [IQR])	2 (2−2)
E component (median [IQR])	2 (1−2)
N component (median [IQR])	3 (2−3)
L component (median [IQR])	2 (1−3)
Preoperative eGFR,mL/min/1.73m2, median [IQR]	70 (60−78)

*IQR* Interquartile range, *BMI* Body mass index, e*GFR* estimated Glomerular fltration rate

The surgical outcomes of RAPN are shown in Table 2. The median operative and warm ischemia times were 232 min (IQR: 194–263) and 20 min (IQR: 17–23), respectively. The median estimated blood loss was 83 mL (IQR: 40–245). We achieved trifecta in 42 patients (84%) and experienced Clavien–Dindo 3A or higher complications in two patients. Both patients developed pseudoaneurysms that were successfully treated using arterial embolization. The median eGFR at POD180 was 65 mL/min/1.73 m^2^ (IQR: 53–71). Significant renal functional decline at POD 180 was observed 19 (38%) of 50 patients.

**Table 2:** Surgical outcomes of radical-assisted partial nephrectomy for cT1b RCC

	n = 50
Trasperitoneal	25 (50)
Retroperitoneal	25 (50)
Operative time, min (median [IQR])	232 (194−263)
Estimated blood loss, mL, (median [IQR])	83 (40−245)
Warm ischemia time, min, (median [IQR])	20 (17−23)
Surgical margin negative, n (%)	50 (100)
Clavien-Dindo ≥ 3	2 (4)
Trifecta achievement, n (%)	42 (84)
Clear	34 (68)
Papillary	6 (12)
Chromophobe	7 (14)
others	3 (6)
eGFR, mL/min/1.73m^2^, (median [IQR])	65 (53−71)
Renal functional decline rate, %, (median [IQR])	9.8 (3.5−17.7)
The number of ≥ 15% reduction of renal function, n (%)	18 (36)

*RCC* Renal cell carcinoma, *IQR* Interquartile range, *BMI* Body mass index, e*GFR* estimated Glomerular fltration rate

**Table 3:** Uni- and multi variable logistic regression analyses for a significant renal functional decline at postoperative day 180

		Significant renal functional decline					
		Univariable			Multivariable		
		OR	95% CI	p value	OR	95% CI	p value
Age	≥ 65 years vs. < 65 years	2.022	0.582−7.027	0.268	1.663	0.266−1.663	0.586
Sex	Male vs. female	2.857	0.817−9.989	0.100			
BMI	≥ 25 vs. < 25	1.368	0.409−4.577	0.611			
Diabetes	Yes vs. no	1.154	0.251−5.300	0.854			
Hypertension	Yes vs. no	0.622	0.195−1.990	0.424			
Dyslipidemia	Yes vs. no	0.823	0.249−2.722	0.750			
Preoperative eGFR	≥ 60 vs. < 60	1.957	0.455−8.421	0.367			
Approach	Transperitoneal vs. retroperitoneal	0.800	0.251−2.551	0.706			
Tumor diameter	≥ 50 mm vs. < 50 mm	1.278	0.367−4.445	0.700			
Warm ischemia time	≥ 25 min vs. < 25 min	4.286	0.700−26.241	0.115			
Estimated blood loss	≥ 200 mL vs. < 200 mL	5.400	1.531−20.382	0.013	9.827	1.647−58.620	0.012
RENAL nephrometry score	≥ 10 vs. < 10	1.79	1.037−3.090	0.037	6.443	0.955−43.3442	0.056
E component	≥ 2 vs. < 2	1.556	0.467−5.182	0.472			
N component	≥ 3 vs. < 3	1.591	0.523−4.845	0.414			
L component	≥ 2 vs. < 2	15.000	1.777−126.596	0.008	15.052	1.331−170.228	0.028

*BMI* Body mass index, e*GFR* estimated Glomerular filtration rate

**Fig. 1:**
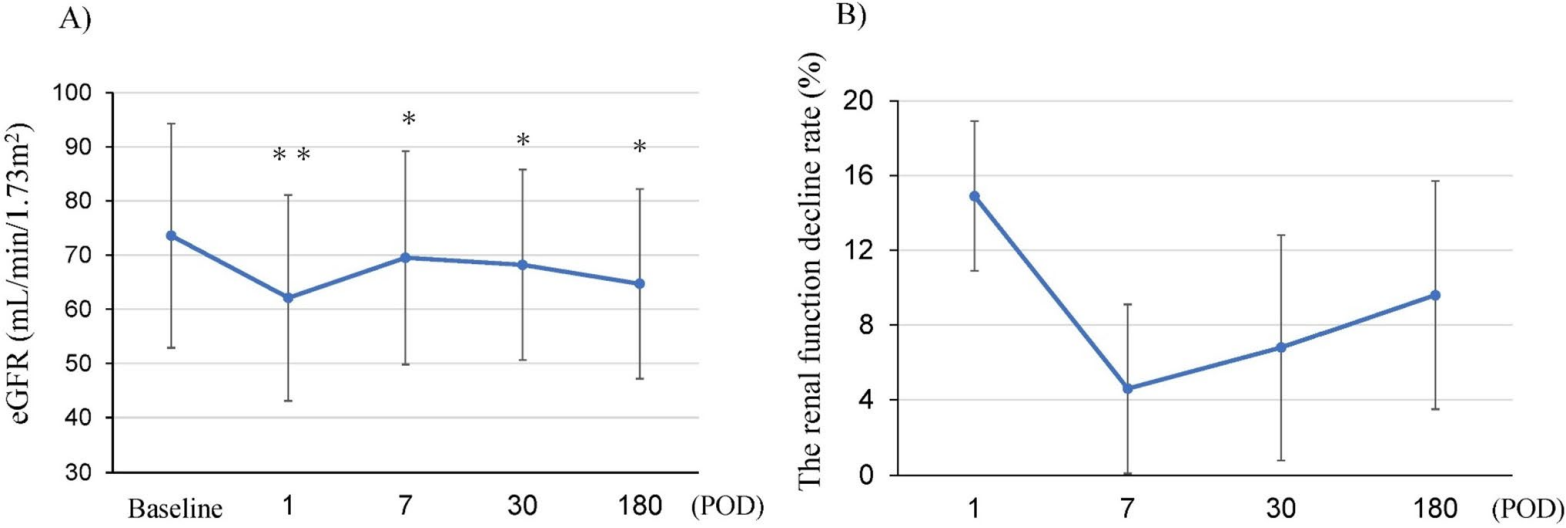
The course of renal function after robot-assisted partial nephrectomy. **A** Change in the mean estimated Glomerular Filtration Rate (eGFR). ⁎*p* < 0.01, ⁎⁎*p* < 0.001. **B** The mean renal function decline rate (%) from Baseline eGFR

Figure 1 shows the course of eGFR and rate of renal functional decline. Renal function declined significantly at POD1 and then improved by POD7. Between POD7 and POD 180, renal function gradually declined.

Univariate and multivariate logistic regression analyses (including age, sex, RENAL nephrometry score, and perioperative factors, such as operative time and estimated blood loss) were performed to elucidate the risk factors for renal function decline (Table 3). In univariate analysis, RENAL nephrectomy score (OR 1.790; 95% CI 1.037–3.090; P = 0.037) and estimated blood loss ≥ 200 mL (OR 5.400; 95% CI 1.531–20.382; P = 0.013) were significant risk factors for renal function decline. Among components in the RENAL nephrectomy score, the L component showed a significant impact on renal function (OR, 15.000; 95% CI 1.777–126.596; P = 0.008). In the multivariate analysis, both L component of the RENAL nephrometry score (OR] 15.052; 95% CI 1.331–170.228; P = 0.028) and estimated blood loss > 200 mL (OR 9.827; 95% CI 1.647–58.620; P = 0.028) were identified as significant risk factors for renal function decline.

## Discussion

In this study, we investigated the factors influencing postoperative renal function after RAPN in patients with T1b RCC. The RENAL nephrometry score is a widely used scoring system for evaluating the complexity of renal tumors and is based on five parameters: tumor size, exophytic/endophytic properties, nearness to the collecting system/renal sinus, anterior/posterior location, and hilar location [12]. Our findings suggested that higher RENAL nephrometry scores, especially higher L components and estimated blood loss ≥ 200 mL were associated with significantly decreasing renal function. This decline is likely caused by the increased difficulty in preserving the renal parenchyma during surgery for tumors with higher RENAL nephrometry scores. Takahara et al. reported that the trifecta rate was significantly lower in patients with higher RENAL nephrometry scores [13]. In patients with high L components in T1b tumors, resection of a large central T1b tumor leads to a greater loss of renal function. This deterioration may be caused by the interference of blood flow to the periphery by the renorrhaphy itself. In addition, in cases of high L-component tumors, it is often necessary to perform substantial resection of the normal renal cortex to secure a safe margin from the tumor, with the potential consequence of renal functional decline. In support of these views, Takagi et al. found that in cases of large, near-central tumors, only 70–80% of the renal cortex could be preserved [14]. Similarly, Kim et al. reported that the tumors with higher L components exhibited more significant postoperative renal function decline and a reduced likelihood of achieving Pentafecta (Pentafecta was defined as the trifecta criterion, > 90% preservation of eGFR, and no stage upgrade of chronic kidney disease at 12 months) [15].

Blood loss during surgery has been identified as an additional factor affecting postoperative renal function. To manage a relatively severe hemorrhage, more sutures are necessary, and additional nephron loss might occur. Too deep or too broad suturing for a hemostat can cause a reduction in blood supply to the remaining kidney parenchyma. Inadvertent and inappropriate suturing may cause a wider ischemic region and subsequent impairment of renal function. Careful intervention is required to minimize blood loss during RAPN and preserve postoperative renal functional outcomes.

Previous reports have indicated that aging, diabetes, large tumors, heavy weight, longer WIT, longer surgery time, and high RENAL nephrometry scores influence renal function after partial nephrectomy [16–19]. Our study did not find a significant association between comorbidities, such as diabetes and postoperative renal function. This may be because of the small number of patients with comorbid diabetes, and our study period may not have been long enough to assess the impact of metabolic disorders on renal function.

Regarding the association between WIT and renal function, a longer WIT was associated with an increased risk of acute kidney injury following partial nephrectomy [20]. In contrast, a WIT less than 30 min does not affect the long-term prognosis of renal function [20, 21]. Similarly, the median WIT was as short as 20 min and did not affect postoperative renal function in the present study. This suggests that careful surgical procedures and avoidance of hemorrhage may minimize the risk of renal function decline.

In this study, we focused on 50 patients with cT1b RCC from the 262 patients with cT1 RCC who underwent RAPN. Around the same time, we also applied other interventions for cT1b RCC. There were 26 and 21 cases of laparoscopic radical nephrectomy (LRN) and open partial nephrectomy (OPN) for cT1 RCC. Of these, specifically for cT1b renal cancer, there were 11 LRN and 8 OPN cases. The surgical procedure for cT1b RCC was determined on each patient. LRN was frequently the preferred approach for elderly patients with well-preserved renal function or for tumors that were potentially classified into T3 based on imaging modalities. In contrast, OPN was selected for patients with a history of multiple abdminal surgeries, (Supplemental Table 1).

According to the 2021 American Urological Association guidelines, patients who do not reach CKD stage 3b (defined as a GFR of 45 mL/min/1.73 m^2^ or less) postoperatively are typically considered suitable candidates for RN. However, only 8% of patients who underwent RAPN reached CKD stage 3b in our cohort, and 50% of LRN deteriorated to stage 3b. This substantial difference underscores the potential of PN to better preserve renal function relative to RN. The findings of this study could be instrumental in guiding the choice between RAPN and LRN in treating cT1b RCC, particularly when considering the preservation of renal function.

The major limitation of our study is its retrospective nature, which may have resulted in selection bias and confounding variables. Additionally, our study was conducted at a single institution, which may have limited the generalizability of our findings.

## Conclusions

Our study suggests that when performing RAPN for T1b RCC, careful consideration of the tumor complexity and surgical technique is essential to minimize the risk of postoperative renal function decline. The RENAL nephrometry score, particularly the L component, can serve as a predictive tool for postoperative renal function. In addition, efforts to minimize intraoperative blood loss may contribute to more efficient preservation of postoperative renal function. Further studies with larger sample sizes and extended follow-up periods to validate our findings and uncover additional influential factors on postoperative renal function are warranted for more efficient RAPN.

## Supplementary Information

Below is the link to the electronic supplementary material.

Supplementary file1 (DOCX 16 KB)

## Data Availability

Raw data were generated at Akita University Hospital. Derived data supporting the findings of this study are available from the corresponding author upon reasonable request.
