# Supplementary material for: Predictive factors of renal function after robot-assisted partial nephrectomy in clinical T1b tumors
Source: J Robot Surg. 2024 Apr 2;18(1):154. doi: 10.1007/s11701-024-01848-3 (PMC10987366; doi:10.1007/s11701-024-01848-3)
Supplement: Supplementary file 1 — Supplementary file1 (DOCX 16 KB) [file 11701_2024_1848_MOESM1_ESM.docx]

| Supplemental Table1. Patient demographics of laparoscopic radical nephrectomy and open partial nephrectomy | | | |
| --- | --- | --- | --- |
|  |  | LRN n = 11 | OPN n = 8 |
| Age, years (median [IQR]) |  | 78 (65 - 86) | 64(55 - 76) |
| Sex, n (%) | Male | 2 (18) | 7 (88) |
|  | Female | 9 (82) | 1 (12) |
| BMI, kg/m2 (median [IQR]) |  | 21.6 (21.0 - 25.7) | 24.5 (20.7 - 32.6) |
| Diabetes mellitus, n (%) |  | 3 (27) | 3 (38) |
| Hypertension, n (%) |  | 6 (55) | 4 (50) |
| Hyperlipidemia, n (%) |  | 5 (45) | 2 (25) |
| Laterality, n (%) | Right | 5 (45) | 5 (62) |
|  | Left | 6 (55) | 3 (38) |
| Tumor diameter, mm (median [IQR]) |  | 44 (35 - 53) | 54 (46 - 60) |
| RENAL nephrometry score (median [IQR]) |  | 8 (7 - 9) | 9 (8 - 10) |
| R component (median [IQR]) |  | 2 (2 - 2) | 2 (2 - 2) |
| E component (median [IQR]) |  | 2 (1 - 2) | 2 (1 - 2) |
| N component (median [IQR]) |  | 3 (2 - 3) | 3 (2 - 3) |
| L component (median [IQR]) |  | 2 (1 - 3) | 2 (1 - 3) |
| Preoperative eGFR  ,mL/min/1.73m2, median [IQR] |  | 71 (61 - 79) | 60 (42 - 92) |
| Postoperative day at 180 eGFR  ,mL/min/1.73m2, median [IQR] |  | 48 (42 - 58) | 47 (31 - 66) |
| IQR interquartile range; BMI body mass index; eGFR estimated glomerular fltration rate | | | |
